# Increase in Mental Disorders During the COVID-19 Pandemic - Role of Occupational & Financial Strains

**DOI:** 10.1093/eurpub/ckac129.028

**Published:** 2022-10-25

**Authors:** N Dragano, M Reuter, A Peters, B Schmidt, B Bohn, K Berger

**Affiliations:** 1 Institute of Medical Sociology, University of Düsseldorf, Düsseldorf, Germany; 2 Institute for Epidemiology, Helmholtz Center Munich, Munich, Germany; 3 Medical Faculty, Ludwig-Maximilians-Universität München, Munich, Germany; 4 Medical Faculty, University Hospital of Essen, Essen, Germany; 5 NAKO e.V., Heidelberg, Germany; 6 Institute of Epidemiology and Social Medicine, University of Münster, Münster, Germany

## Abstract

**Background:**

Numerous studies reported an increase in mental disorders during the COVID-19 pandemic, but the specific causes for this increase are unclear. We therefore investigate whether pandemic-related occupational and financial changes (e.g., reduced working hours, working from home, financial losses) were associated with increased symptoms of depression and anxiety compared with the situation before the pandemic.

**Methods:**

We analyzed data from the German National Cohort Study (NAKO). Between May and November 2020, 161,849 participants answered questions on their mental state and social circumstances. Responses were compared with data from the baseline survey before the pandemic (2014-2019). Linear fixed-effects models were used to determine whether individual changes in the symptoms of depression (PHQ-9) or anxiety (GAD-7) were associated with occupational/financial changes (controlling for covariates).

**Results:**

A pronounced increase in symptoms was observed among those who became unemployed during the pandemic (+ 1.16 points on the depression scale, 95% confidence interval [0.91; 1.41], range 0-27). Increases were also seen for reduced working hours without short-term working allowance, increased working hours, working from home, insecurity regarding employment, and financial strain. The deterioration in mental health was largely statistically explained by the occupational and financial changes investigated in the model.

**Conclusions:**

Depressive symptoms and anxiety disorders increased in the study population during the first year of the COVID-19 pandemic and occupational and financial difficulties were an essential contributory factor. These strains should be taken into account both in the care of individual patients and in the planning of targeted prevention measures. Results suggest that welfare state benefits such as short-time allowance in times of crises may reduce mental load in affected populations.

